# DNA polymerases engineered by directed evolution to incorporate non-standard nucleotides

**DOI:** 10.3389/fmicb.2014.00565

**Published:** 2014-10-31

**Authors:** Roberto Laos, J. Michael Thomson, Steven A. Benner

**Affiliations:** Foundation for Applied Molecular EvolutionGainesville, FL, USA

**Keywords:** DNA polymerases, non-standard nucleotides, AEGIS, directed evolution, CSR, protein engineering

## Abstract

DNA polymerases have evolved for billions of years to accept natural nucleoside triphosphate substrates with high fidelity and to exclude closely related structures, such as the analogous ribonucleoside triphosphates. However, polymerases that can accept unnatural nucleoside triphosphates are desired for many applications in biotechnology. The focus of this review is on non-standard nucleotides that expand the genetic “alphabet.” This review focuses on experiments that, by directed evolution, have created variants of DNA polymerases that are better able to accept unnatural nucleotides. In many cases, an analysis of past evolution of these polymerases (as inferred by examining multiple sequence alignments) can help explain some of the mutations delivered by directed evolution.

## INTRODUCTION

DNA polymerases are enzymes that catalyze the template-directed synthesis of DNA. Over billions of years, they have evolved to have the speed, specificity, and accuracy required for them to transmit valuable genetic information to and from living organisms with a level of infidelity just sufficient to support Darwinian evolution.

Currently, many DNA polymerases are used in polymerase chain reaction (PCR) and other procedures that involve the copying of nucleic acids. These include multiplexed PCR, nested PCR, reverse transcription PCR, and DNA sequencing. Polymerases are also used to incorporate modified nucleotides, including those that tag, report, or signal the presence of product DNA molecules. They are also now being used to copy sequences built from “artificially expanded genetic alphabets,” which add new base pairs to the standard A:T and G:C pair. Together, these technologies are combined to allow nucleic acids to be amplified from complex samples, including saliva, blood, forensic traces, and fossil remains. Furthermore polymerases are supporting *in vitro* selection with expanded genetic alphabets to create receptors that bind to cancer cells (Sefah et al., 2014). Accordingly, the demand for new polymerase variants, especially those with specialized attributes, shows no sign of diminishing, despite the large number of polymerases already available.

This review focuses primarily on polymerase variants that accept nucleic acids having additional nucleotide “letters” that form additional nucleobase pairs. Such expanded genetic systems are being developed in many laboratories (Rappaport, 1988; Switzer et al., 1989; Ishikawa et al., 2000; Tae et al., 2001; Kool, 2002; Geyer et al., 2003; Henry and Romesberg, 2003; Minakawa et al., 2003; Benner, 2004; Hirao et al., 2004; Sismour and Benner, 2005). Some of these simply shuﬄe the hydrogen bonding groups that join base pairs within a Watson-Crick geometry, such as the artificially expanded genetic information system (AEGIS; Piccirilli et al., 1990; Geyer et al., 2003). Others attempt to add hydrogen bonds to hold the pair together (Minakawa et al., 2006). Still others hope to dispense with hydrogen bonds entirely (Morales and Kool, 1999). Some polymerases have been modified without the use of directed evolution; however, these cases provide an insight on structure and function of polymerases.

Major advances in “next generation” sequencing, which requires the use of modified nucleotides and DNA polymerases, are considered in a separate review in this series (Chen, 2014).

## DNA POLYMERASE FAMILIES

DNA polymerases have been classified into evolutionary families based on an analysis of their amino acid sequences. Initially two decades ago Braithwaite and Ito (1993) used an extensive compilation of the then-available sequences to classify polymerases into three families: A, B, and C. The family names indicated homology to the products of three genes: *pol*A, *pol*B, and *pol*C, which encode for the three canonical polymerases from *Escherichia coli*: DNA polymerase I, DNA polymerase II and DNA polymerase III alpha subunit, respectively (Ito and Braithwaite, 1991; Braithwaite and Ito, 1993). The most studied polymerases belong to Family A (found in prokaryotes, eukaryotes and bacteriophages) and family B (found in prokaryotes, eukaryotes, archaea, and viruses). The D family groups polymerases from Archaea (Cann and Ishino, 1999). Families X and Y are involved in repair. The family X perform base excision repair and double-strand break repair by using their ability to fill short gaps (Moon et al., 2007; Yamtich and Sweasy, 2010). Some polymerases from the family X can perform polymerase activity without template (Berdis, 2014). The family Y groups eukaryotic polymerases (Ohmori et al., 2001) and these show less homology to the previously identified families. Most of family Y polymerases lack proofreading exonuclease domains and have a more open active site to accommodate base damage, presumably this allows them to bypass DNA lesions (Pryor et al., 2014). The RT family groups the reverse transcriptases, including eukaryotic telomerases and reverse transcriptases found in viruses (Le Grice and Nowotny, 2014).

Early studies recognized that mild proteolysis of DNA polymerase I from *E. coli* produces two fragments, a large fragment that lacks the 5′–3′ exonuclease activity and a small fragment that is then discarded. The large fragment, called the Klenow fragment, retains both the polymerization and proofreading activities of the native enzyme. The Klenow fragment yielded the first crystal structure of a family A polymerase, solved by Ollis et al. (1985). This crystal revealed a “right hand” shape with the active site being located at the “palm” which holds the catalytic amino acids, a “thumb” that binds double-stranded DNA and “fingers” where the incoming nucleotide binds and interacts with the template. The structure of *Thermus aquaticus* DNA polymerase and the analog of the Klenow fragment, the large fragment of *Thermus aquaticus* DNA polymerase (Klentaq1) has also been studied by crystallography (Kim et al., 1995; Korolev et al., 1995).

### FAMILY A POLYMERASES

Family A is the most studied of the seven DNA polymerase families. It includes many of the “workhorse” polymerase in classical molecular biology, including the Klenow fragments of *E. coli* and *Bacillus* DNA polymerase I, *Thermus aquaticus* DNA polymerase and the T7 RNA and DNA polymerases. It also includes the first DNA polymerase to be characterized enzymatically, DNA polymerase I from *E. coli*, in seminal work by Kornberg (1960).

#### *Taq* polymerase

With the advent of the PCR, it became clear that a polymerase stable to heating would be useful. Here, the DNA polymerase I from *Thermus aquaticus* (*Taq* polymerase) is widely used in PCR. *Thermus aquaticus* was isolated in 1976 from hot springs in Yellowstone National Park (Chien et al., 1976), where it thrives at 70° C. Since the enzyme can be activated by heating the sample and remains active with the high temperatures required to denature DNA strands (typically 94°C), it allows repeated cycles of denaturing, annealing and extension (thermocycling) without the need to add additional polymerase at each cycle. This made PCR a routine laboratory technique.

Eom et al. (1996) solved a co-crystal structure of *Taq* with blunt DNA duplex bound to the active site cleft. This structure had several features: (a) DNA is in an intermediate form between B and A forms. (b) Functionality from certain amino acid side chains hydrogen-bond to the *N*^3^ of purines and the *O*^2^ of pyrimidines of specific residues in the duplex. (c) The 3′ hydroxyl of the primer strand is near three carboxylate groups, delivered by amino acids Asp 785, Glu 786, and Asp610. These are considered to constitute the catalytic core of the enzyme.

As with its homolog, polymerase I from *E. coli*, Taq DNA polymerase can be cleaved to give an active fragment, called Klentaq. This fragment retains polymerase activity without one of its nuclease activities. Li et al. (1998) solved the crystal structures of two ternary complexes of the large fragment of *Thermus aquaticus* DNA polymerase I (Klentaq1): (a) Klentaq with primer/template and dCTP; (b) Klentaq with primer template. These identified two conformations of the polymerase: (i) an “open” conformation where the tip of the fingers of the hand is rotated 46° outward and presumably not actively performing the polymerase reaction and (ii) a “closed” conformation, which is “caught in the act” of incorporating a nucleotide. This was the first direct evidence in any DNA polymerase for a large conformational change as part of the catalytic cycle.

#### Motifs relevant to the engineering of Family A polymerases

Six motifs in the structure of *Taq* Pol are also conserved throughout Family A polymerases. These include motifs A, B, and C (Delarue et al., 1990). Motifs A and B are the most conserved. These two motifs are relevant to DNA polymerase fidelity and substrate specificity, which makes them of special interest to experimentalists seeking to improve the ability of investigators to obtain polymerases that accept unnatural substrates.

Motif A is found in the palm domain of the polymerase and includes the amino acids 605–617; in *Taq*, the sequence is (LLVALDYSQIELR). Within this motif, Asp 610 (bold D) cannot be changed without losing enzymatic activity, presumably, because it coordinates the metal that is directly responsible for catalysis. Glu 615 (bold E) can be changed to Asp without complete loss of activity. Tyr 611(bold Y) which is located in a hydrophobic pocket, can be replaced by a planar aromatic amino acid. The rest of the amino acids in motif A can be replaced by many amino acids without destroying catalytic activity (Patel and Loeb, 2000a).

**FIGURE 1:**
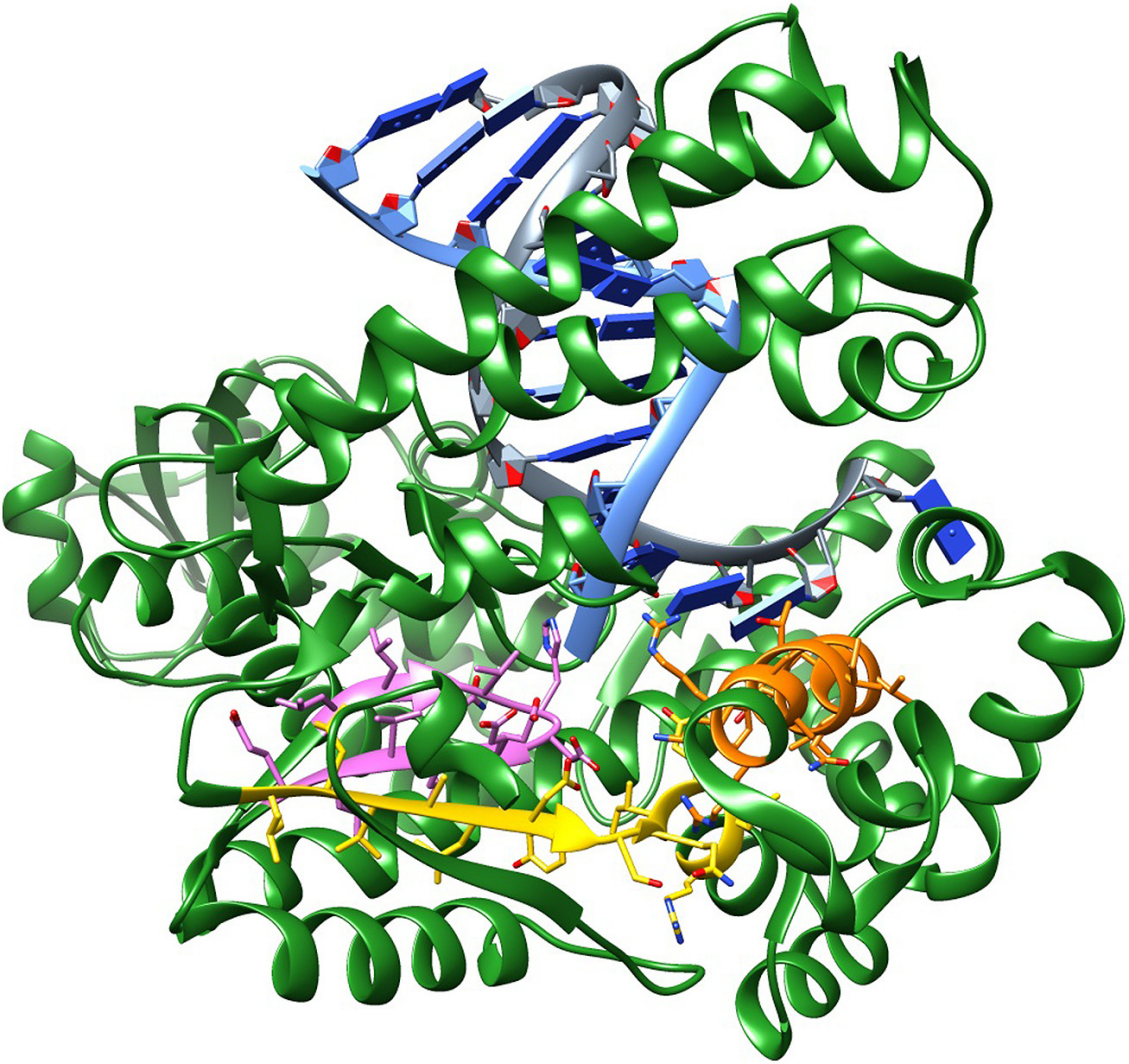
**Structure of *Taq* DNA polymerase showing the conserved motifs: **(A)** (LLVALDYSQIELR) – yellow; **(B)** (RRAAKTINFGVLY) – orange; **(C)** (LLQVHDELVLE) – pink.** PDB ID 4DLG, which includes *Taq* beginning from the RNaseH portion till the end, in complex with a DNA primer and a DNA template, halted by/at a ddCTP.

The amino acids that make up motif B are located in the fingers domain and form the O-helix, which contacts the base pair being formed in the primer extension step. This motif covers residues 659–671; in *Taq*, the sequence is (RRAAKTINFGVLY). This motif contains Arg 659 (bold R) and Lys 663 (bold K), which are known to interact with the incoming triphosphate moiety and are critical for enzymatic activity. For these reasons, they are most likely immutable. Alternatively, Phe 667 and Tyr 671 tolerate conservative substitutions as these are involved in base stacking. The remaining amino acids tolerate a wide range of substitutions. **Figure 1** shows the conserved motifs on the structure of *Taq* polymerase.

Certain substitutions within motif A and B have shown to lower fidelity without eliminating catalytic activity (Suzuki et al., 1997). This is the case of Ile614 in motif A and the Ala661Glu and Thr664Arg substitutions on motif B.

In one example, replacing an amino acid at a single site is known to change substrate specificity in a useful way. Replacement of Phe667 in *Taq* polymerase by a tyrosine eliminates the ability of the polymerase to discriminate against dideoxynucleotides. The *Taq* variant F667Y is, therefore, used for DNA sequencing. Interestingly, in T7 DNA polymerase, the replacement of Tyr526 by Phe increases the discrimination against dideoxynucleotides. This illustrates a general principle in protein engineering: rationales are best constructed *after* the replacement is made and its impact is evaluated.

### FAMILY B POLYMERASES

Exploration of the natural microbiosphere led to the discovery of organisms that could grow at temperatures even higher than *Thermus*. These came to be known as “hyperthermophiles,” and were shown by their ribosomal RNA sequences to belong to a third kingdom, or domain, of life of Earth: the Archaea. These proved to be sources of polymerases that were even more thermostable.

For example, *Pyrococcus furiosus* (*Pfu*), a hyperthermophilic archaeon, was discovered in the Lower Geyser Basin of Yellowstone National Park (Brock and Freeze, 1969; Brock and Edwards, 1970). Its DNA polymerase (*Pfu*) has been used in many PCR applications. Crystallographic analysis of the native form (Kim et al., 2008) as well as a variant able to replace dCTP with a cyanine dye-labeled dCTP (Wynne et al., 2013) showed that it contains five distinct domains, called the finger, palm, thumb, N-terminal and exonuclease domains (Hopfner et al., 1999; Hashimoto et al., 2001).

*Pyrococcus furiosus* has a feature that is absent in *Taq* polymerase: an exonuclease domain that has 3′–5′ exonuclease activity. This allows *Pfu* to proofread using a conformational change (Hopfner et al., 1999). When the polymerase encounters a mismatch, it binds more weakly to the primer/template, causing strand unwinding. This allows the mismatch to move into the exonuclease pocket, where excision ensues (Freemont et al., 1988). A conserved loop in the exonuclease domain interacts with the thumb domain (Kuroita et al., 2005). Mutation of a key residue H147 to a glutamate residue in this loop results in an electrostatic attraction of the thumb domain to the exonuclease domain, preventing the 3′ end of single stranded DNA from entering the exonuclease domain, thus significantly reducing the 3′–5′ exonuclease activity (Wang et al., 1997; Kuroita et al., 2005). Kim et al. (2008) suggested that an alternative residue E148 was located at a better position in the loop to interact with the thumb domain through a comparison of the crystal structures of *Pfu* and KOD1, a homologous Family B polymerase from the *Thermococcus* genus.

Family B DNA polymerases of hyperthermophilic archaeons may generally have 3′–5′ exonuclease activity (Joyce, 1989; Joyce and Steitz, 1994; Benkovic et al., 2001; Joyce and Benkovic, 2004). Some have shown to recognize the presence of uracil and hypoxanthine in a template strand, stalling when they sense it ahead of the extension site (Greagg et al., 1999; Fogg et al., 2002; Connolly, 2009). This may reflect functional adaptation. Both uracil and hypoxanthine are “mistakes” in a DNA sequence, arising via the deamination of cytosine and adenine, respectively. Such deaminations presumably occur more rapidly at the higher temperatures where hyperthermophiles live. If the polymerase, nevertheless, extends further through the incorporation of dNTPs placing the uracil in the +2 position, the resulting outcome is the activation of the proofreading excision of the deaminated base (Connolly, 2009).

The need for additional proofreading in the natural environment may not be so pressing to a biotechnologist. Accordingly, many have altered the proofreading ability of *P. furiosus* by either removing the exonuclease activity for use in error-prone PCR (ePCR; Biles and Connolly, 2004) or increasing the efficiency of ligation-mediated PCR protocols (Angers et al., 2001). Sanger sequencing also requires the elimination of the exonuclease activity, otherwise incorporated ddNTPs would be removed and the sequencing signals would disappear.

## PROTEIN ENGINEERING AND DIRECTED EVOLUTION

### THE “NEXT GENERATION” GOALS

For classical Sanger sequencing, a polymerase need only accept a tagged triphosphate with a 3′-blocking group with modest fidelity. The termination:extension ratio will be adjusted in any case by adjusting the concentrations of the terminating and non-terminating triphosphates, meaning that relative inefficiency of incorporation of the unnatural species is not problematic.

However, as the synthetic biology research paradigm has developed, the demands placed on polymerase performance have increased. Here, polymerases are often called upon to copy DNA and PCR amplify molecules containing unnatural nucleotides, often at multiple sites. Here, the fidelity and (preferably) processivity required by a DNA polymerase to support PCR with unnatural nucleotides must be very high. In addition, the structural differences between a DNA polymerase that makes one error per thousand nucleotides and one error per million can be quite subtle and can arise through geometric differences that would not be necessarily distinguished even in a high resolution crystal structure.

### RATIONAL DESIGN

#### Information from structural biology

Molecular biologists would like to believe that they have command of structural theory to “rationally” design polymerases with new, anticipatable properties. In some cases, this is possible, especially when it involves domain shuﬄing. This has been productive in improving one feature of polymerases important for a wide range of applications: processivity.

DNA binding factors are known to enhance processivity of many polymerases charged with copying complete microbial genomes. In principle, these might be added to improve the processivity of Pol I polymerases, which (as noted above) do not perform this role naturally. Their addition might also, in principle, be used to enhance the performance of any polymerase or polymerase variant. This addition, however, is not often used in biotechnology because of the complexity of the assembled combination. Indeed, *Taq* polymerase and other enzymes are used without accessory proteins for PCR because of their simplicity, which comes from their physiological roles in lagging strand replication and DNA repair.

The complexity of a multicomponent system would be avoided by directly fusing a processivity domain to the active polymerase domain. Adopting this rationale, Wang et al. (2004) covalently fused the double stranded DNA binding protein Sso7d from *Sulfolobus solfataricus* at the N-terminus of *Taq* polymerase (S-*Taq*) and to the fragment of Taq polymerase that results from the deletion of the first 289 amino acids which lacks the exonuclease domain [S-*Taq*(Δ289)]. The average length of primer extension prior to template-primer dissociation with *Taq* (Δ289) was increased from 2.9 to 51 nucleotides in S-*Taq*(Δ289). The full-length *Taq* polymerase, which is intrinsically more processive than *Taq* (Δ289), improves its average primer extension from 22 (*Taq*) to 104 (S-*Taq*) nucleotides (Wang et al., 2004).

In parallel work, Wang et al. (2004) also fused the Sso7d domain to the C-terminus of the polymerase from *P. furiosus*, to give *Pfu* polymerase (*Pfu*-S). As in the case of *Taq* polymerase, the fusion of the Ssod7 domain lead to an increase of the average primer extension, from 6.4 nucleotides for *Pfu* to 55 for *Pfu*-S.

Uses for the more processive (*Pfu*-S) were further realized in 1999 when the crystal structure of *Thermococcus gorganarius* DNA polymerase (*Tgo*) was solved. This structure identified a uracil binding pocket, which is used physiologically to prevent the polymerase from copying a template containing uracil, which arises from deamination of cytosine. This structure directed the construction of mutant forms of *Tgo* and *Pfu* DNA polymerases with reduced uracil stalling (Hopfner et al., 1999; Fogg et al., 2002). To increase the ability to read through uracil in the template, the Ssos7 domain was fused to both (*Pfu*-S) and the high fidelity mutant *Pfu* (V93Q). The result was higher processivity and improved uracil-excision cloning (Nour-Eldin et al., 2006).

Structural biology also provided a domain-swapping rational to increase the processivity of *Taq* polymerase. Here, the thioredoxin binding domain (TBD) of the T3 bacteriophage DNA polymerase was inserted into the thumb domain of *Taq* DNA polymerase, deleting amino acids 480–485 (Davidson et al., 2003). The rationale recognized that the processivity of T7 DNA polymerase increases from 15 to 2000 nucleotides when it forms a complex with *E. coli* thioredoxin. The affinity to the primer-template is also increased 80-fold upon binding to thioredoxin. The polymerase arising from this domain fusion remains thermostable, and has a 20–50 times higher processivity than the original *Taq* polymerase.

#### Exploiting information from multiple sequence alignments (MSAs) in rational engineering

Polymerases are, of course, widely distributed in the biosphere in homologous form. During their divergent evolution, natural selection superimposed upon random variation has carried out several billion years of “protein engineering” experiments, of a sort. With the explosion of microbial sequencing in the last two decades, the results of these “experiments” can be obtained from a public sequence database. To the extent that these results are not corrupted by sequence error, they provide “evolutionary guidance” to assist laboratory protein engineering (Weinhold et al., 1987).

Evolutionary guidance has been productively applied to engineer polymerases, with Tabor and Richardson (1995) providing a classic example. Seeking to improve the ability of *Taq* DNA polymerase I to accept 2′,3′-dideoxynucleoside triphosphates (ddNTPs) for sequencing applications, Tabor and Richardson (1995) examined the sequences of three DNA polymerases from Family A (Braithwaite and Ito, 1993). “Wet” biochemistry had already told them that one of these, that from bacteriophage T7, incorporated ddNTPs better than the two others, polymerases from *E. coli* and *Thermus aquaticus*.

Tabor and Richardson (1995) then constructed a multiple sequence alignment (MSAs) for the three homologous Family A polymerases. They noticed that T7 polymerase had a tyrosine at a site (numbered 526) that is homologous to positions that held a phenylalanine in the *E. coli* and *Taq* polymerases (numbered 762 and 667 respectively). From this comparison, they hypothesized that this single amino acid difference was responsible for the different levels of discrimination against ddNTPs among the three polymerases.

Based on this hypothesis, Tabor and Richardson (1995) replaced the phenylalanine in the *Taq* polymerase by a tyrosine. The result was a variant *Taq* (F667Y) that retained the thermostability of the *Taq* parent but gained improved ability to accept ddNTPs. Similar improvements were seen when the analogous replacement was made in the polymerase from *E. coli*. The mutant *Taq* (F667Y) became one of the first “designed” polymerases to be used in DNA sequencing (Tabor and Richardson, 1995).

Subsequently, Li et al. (1999) studied the crystal structures of Klentaq1, a derivative of *Taq* DNA polymerase that lacks an exonuclease domain. In separate structures, protein crystals binding ddNTPs were observed to have closed ternary complexes, where a conformational change upon substrate binding was associated with a large shift in the position of the side chain of residue 660 in the O helix. Comparing the open and closed structures with ddGTP, Li et al. (1999) concluded that the selective interaction of arginine 660 with the *O*^6^ and *N*^7^ atoms of the G nucleobase might provide structural grounds for better incorporation of ddGTP by *Taq* polymerase. Guided by these observations, Li et al. (1999) then replaced amino acids at residue 660 in Klentaq1 already holding the Tabor-Richardson replacement (F667Y) and studied the resulting variants. Among the variants, the double mutant *Taq* (F667Y; R660D) showed superior performance in DNA sequencing architectures that used ddNTPs.

### THE NEED FOR DIRECTED EVOLUTION: SEQUENCE LANDSCAPES

While structure, evolutionary comparison, and mechanistic analysis are all important tools in polymerase engineering, it remains a fact that chemical theory is inadequate to predict the exact outcome of any amino acid replacement on the performance of any protein, including polymerases. A degree of “trial and error” is inherent in protein engineering experiments. This, in turn, requires that we consider the size of the “protein sequence space” that might be explored as we set out to modify a protein to allow it to support a specific technological goal.

Background to this concept was presented by Smith (1970) almost a half century ago. We begin by noting that the behavior of all possible proteins of length *n* with respect to a measurable behavior can be represented by a space in *n* dimensions, where each dimension can have one of 20 discrete values, representing the 20 natural amino acids. Each protein sequence is represented by a point in that space. Two points are neighbors in that space if one can be converted into another by a single amino acid substitution. Thus, with 20 amino acids, each point in the sequence space has 19*n* neighbors. The measurable behavior is a real number displayed in the *n*th +1 dimension.

**FIGURE 2:**
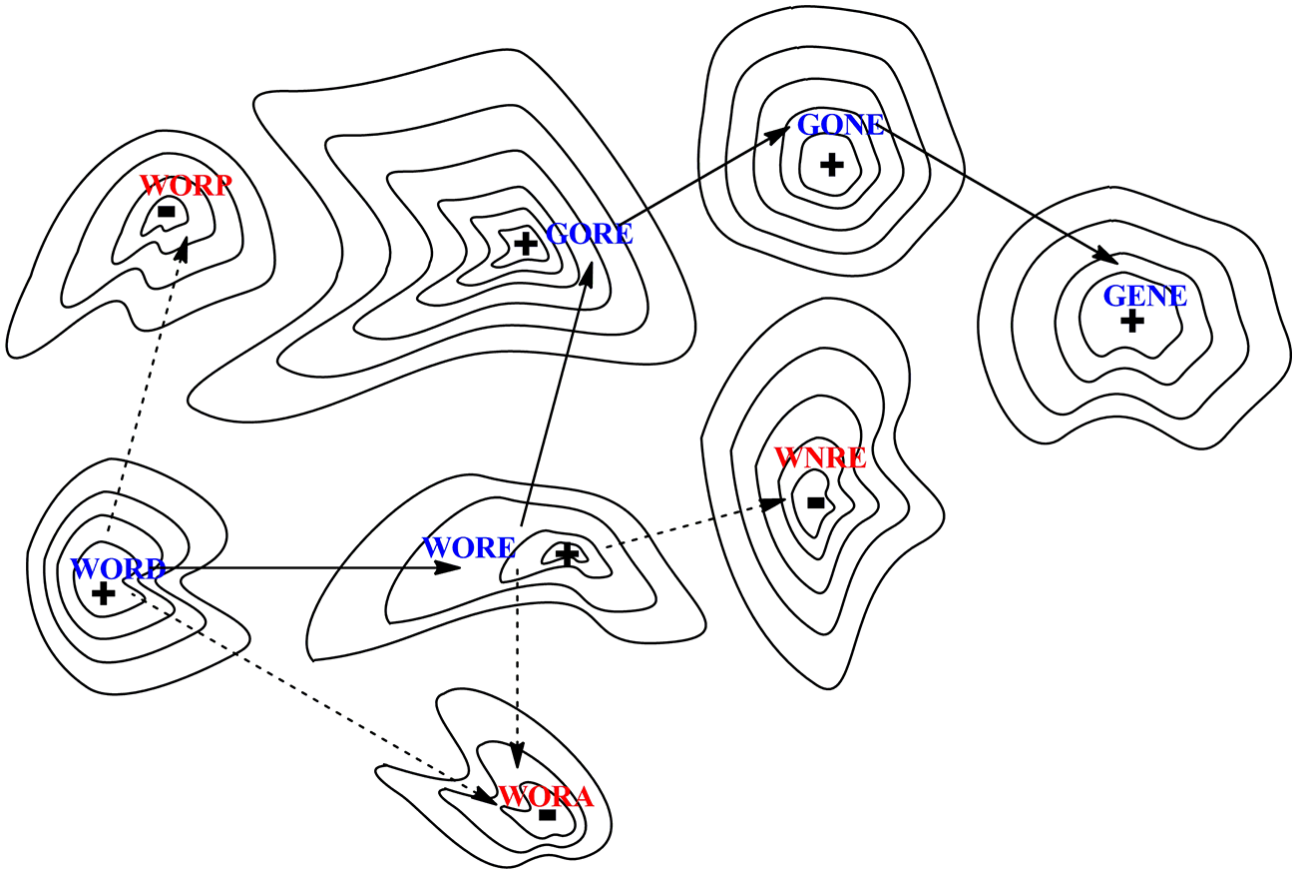
**Evolution can be modeled as a walk across a fitness landscape, here presented as a two-dimensional representation of a multiple dimensional hypersurface; analogous to a topographic map, peaks (+) indicate the locations where function exist while dips (–) represent regions with lack of function.** Illustrated through an analogy to a word game, a meaningful (functional) string of letters (here “word”) must be reached starting from another string (“gene”) via stepwise replacement of single letters, where every intermediate along the path must itself also be a functional word. Solid arrows indicate a path of accepted mutations while dashed arrows illustrate deleterious mutations that produce non-functional proteins.

Different sequences have different functions, and moving from a sequence having a function to another functional sequence can proceed via intermediates that either have or lack function. This is illustrated in **Figure 2** with a word game used by Smith (1970), where functional protein sequences are analogous of strings of letters that have a meaning in English. In Smith’s (1970) analogy, the sequence of letters in the word “WORD” is converted to the sequence of letters in the word “GENE” by exchanging one letter at the time, with each step in one path having a meaning (WORE, GORE, and GONE). Paths where all intermediates are meaningful are illustrated by solid lines between points on the surface. Other paths proceed via words lacking meaning, as illustrated by broken lines (for example, WOND, GOND, and GEND).

In this example, linguistic “meaning” is equated to fitness, which provides the *n*th +1 dimension to the surface, a “fitness landscape” (Wright, 1932). The landscape is represented as a topographic map with peaks marked with a (+) for optimal sequences. The absence of function is depicted as dips, marked with a (-). Smith (1970) proposed that natural evolution evolves along paths only if all intermediates are functional. Non-functional sequences are removed by “purifying” selection. Thus, the only valid pathways to explore a sequence space proceed via functional sequences, just as the evolution of words can proceed only via meaningful words.

Sequence space within a protein framework is vast, but enumerable. For example, a 100-amino acid protein can be arranged in 20^100^ different ways. Typical polymerases, eight times longer, constitute a space with 20^800^ points. Both numbers are astronomical. No experiment can sample this space effectively.

Several features of the fitness landscape influence the ease with which it is searched: (a) the fitness landscape is “smooth,” meaning that a useful protein sequence can be obtained starting at any point on the landscape via a path that encounters only other functional proteins or, if not, then (b) useful functional proteins can be obtained no matter where one starts the search, as the surface has many of them or, if not, then (c) the library is guided so as to start the search in a region of the functional hypersurface where useful functional proteins reside. Directed evolution is an approach that mimics natural evolution in a time scale that can be reproduced in a laboratory. A directed evolution experiment starts by producing a library of variants (to be discussed further) which then would be selected to a screen or to a selection. A screen involves testing individual variants for the desired properties and is suitable for relatively small libraries, perhaps no more than a few 100s. A selection typically sorts millions of variants at the same time. The experimenter designs the selection in a way that only the variants with the desired properties would survive the selection. The expected outcome of a directed evolution experiment is an enriched pool of variants with proteins having the desired characteristics. Directed evolution can be used to optimize and study any protein (Sterner, 2011).

### THE PRACTICE OF DIRECTED EVOLUTION WITH POLYMERASES

In a directed evolution experiment, a “parent” enzyme is chosen to start the search that has (at least) some of the properties desired in the enzyme that will ultimately have utility. The gene of this parent enzyme is then altered to create a library encoding variant forms of the enzyme; some of which might be able to catalyze the desired transformation better than the parent enzyme. The members of the library that are of interest can be isolated by screening or selection.

#### Library generation

In fact, we have little information about the “smoothness” of any protein fitness landscape. The native polymerase used to initiate an experiment in directed evolution is, of course, already at an elevated point on the fitness landscape, at least for some conditions. It is not clear how many steps (amino acid replacements) can be taken away from the native sequence without losing activity. Further, we expect that certain replacements are more likely to retain core activity than others. All of this suggests that the nature of the library generated from that native sequence might influence the outcome of a directed evolution experiment. It is certainly expected that library generation, if intelligently biased, will allow desired outcomes to be generated faster.

***Error prone PCR.*** A common way to generate libraries from a starting sequence is “mutagenic” or “ePCR.” This approach takes advantage of the inherent propensity of *Taq* polymerase to introduce mistakes into the copies of DNA under certain conditions. The frequency of mismatching is often increased by introducing manganese Mn^2+^ along with the natural cofactor Mg^2+^ (Vartanian et al., 1996). Other additives, such as alcohols or unbalanced concentrations of nucleotides, can also be used to introduce mutations through PCR.

The ePCR method produces does not produce a truly random set of amino acid replacements, for several reasons:

(i) *Taq* pol tends to replace purines (adenine and guanine) by other purines and pyrimidines (thymidine and cytidine) by other pyrimidines; these changes are called transitions (as opposed to transversions, which exchange a purine for a pyrimidine or a pyrimidine for a purine). The biased tendency of the polymerase to generate transitions over transversions leads to libraries with amino acid replacements that are non-random with respect to the parent protein.
(ii) Even if ePCR introduced transitions and transversions equally, the resulting amino acid replacements would not be random, due to the structure of the genetic code. In the code, amino acids having similar chemical properties have closely related codons (Wong et al., 2007). For example, the valine codon (GTN^1^) is converted by a single nucleotide replacement to a phenylalanine codon (TTY^2^), a leucine codon (CTN), an isoleucine codon (ATN), an aspartate codon (GAY) or a glycine codon (GGN). To gain access to codons for other amino acids and, consequently, more dramatically, alter chemical properties in the variant protein, two or three nucleotide replacements are required.

High levels of replacement are not easily achieved by ePCR, nor are they desired. Typical ePCR introduces no more than 4–6 mutations per 1000 nucleotides. Further, a mutation rate that is high enough to search amino acid sequences independent of the code is almost certainly too high to generate any variants that retain polymerase activity as polymerases.

***Degenerate codons.*** Recognizing this challenge, Reetz et al. (2008) developed an elegant approach to library generation that introduces degenerate codons: NNK and NDT. Here, N is any nucleobase, K is guanine or thymine, and D is guanine or adenine or thymine. With the NNK degenerate codon, all 20 amino acids are covered by just 32 (= 4 × 4 × 2) of the 64 codons possible with standard nucleotides. The twelve NDT degenerate codons ( = 4 × 3 × 1) cover a representative sample of the standard amino acids, including non-polar, aromatic hydrophobic, hydrophilic, and charged amino acids.

Behind this discussion are assumptions about the meaning of the word “random” when discussing amino acid replacements. Some amino acids are encoded by more codons than other amino acids, like serine, with six codons; in contrast, tryptophan is encoded by just one codon. A gene with a truly random sequence would give proteins with a codon-weighed distribution of amino acids. Even this might not be the desired goal of an unguided approach to library generation as some amino acids appear in natural proteins more abundantly than expected from their few codons, for example aspartate and glutamate, each with two codons. Thus, an “ideal” library might arguably be one in which amino acids are replaced by a process that leaves the naturally observed overall composition of the protein unchanged. Finally, our ignorance on the shape of function landscapes, as well as our ignorance of the local topography around any individual parent sequence, means that we cannot state *a priori* which amino acid distribution is most likely to give a desired result in a directed evolution experiment.

***Libraries made by gene shuﬄing or molecular breeding.*** Random mutagenesis of a parent gene fails, of course, to use all of the information available to a protein engineer, especially in a post-genomic world. As noted above, Nature has already run evolution experiments. These provide to us many homologs of a parent protein having many amino replacements relative to the parent sequences. Most of these are functional, and, therefore, identify points in sequence space that are elevated on the fitness landscape. It would be desirable to use the information that these homologs provide.

Gene shuﬄing was introduced by Stemmer (1994) more than a decade ago to directly use these homologs. Here, the starting point is a family of genes that share enough sequence similarity that they can undergo homologous recombination. Using a modified PCR protocol, gene chimeras are produced.

Those using shuﬄing in protein evolution assume, of course, that sequence space is more efficiently searched by combining the outcomes of two historically successful searches of a particular region of sequence space, than a search that simply replaces single amino acids starting from a single parent. These historical searches delivered the two functioning proteins whose genes are being shuﬄed. Here, the landscape is assumed to be such that specific paths between two elevated points are also similarly elevated.

This would be a more compelling hypothesis if natural evolution were observed to use shuﬄing. Natural evolution does, of course, have access to mechanisms that shuﬄe parts of genes. Natural evolution uses these mechanisms to rearrange (for example) the order of independently folded units in multi-unit polypeptides. This is famously done in the evolution of multi-unit proteins involved in metazoan signal transduction, where a regulatory protein might contain one “src homology domain 1” unit (SH1, a protein kinase), a few SH2 units, and a few SH3 units (Benner et al., 1993). Evolutionary analysis shows that these are all obtained by shuﬄing, implying that shuﬄing is an efficient way to search sequence space when no protein folding unit is disrupted.

However, natural evolution does not provide many examples where polypeptide chains within a *single* folded unit are shuﬄed. This is presumably because the buried contacts binding collections of secondary structural units are finely tuned to permit packing. Changing a single hydrophobic side chain in a packed protein fold often converts a core that is (typically) as densely packed as an organic crystal into a “molten globule.” Thus, these biophysical realities would make it surprising to expect that shuﬄing explores sequence space more effectively than point mutation. Such expectations rely, of course, on the view that natural evolution exploits the most effective ways to search sequence space.

***Use of evolutionary information to create smaller but better libraries.*** Alternative approaches now exist to create libraries that search sequence space around parent sequences (Lutz and Patrick, 2004; Jackel et al., 2008; Lutz, 2010). One class of these exploits evolutionary guidance. For example, Cole and Gaucher (2011) introduced an approach, called the Reconstructing Evolutionary Adaptive Paths (REAP) to create libraries that were hypothesized to explore local sequence space with more efficiency. REAP begins with a phylogenetic analysis of homologous sequences, seeking signatures of functional divergence. An amino acid at a site may be entirely conserved in one branch of a phylogenetic tree, while not conserved at all in a second branch. This pattern of divergence, sometimes called *heterotachy*, indicates that the purifying selective pressures operating in the first branch at this site are different and stronger than those in the second. This, in turn, means that the function of the proteins within the first phylogenetic branch is different from the function in the second branch.

Only rarely, however, has natural history sought a phenotype desired by a protein engineer, of course, only rarely responsive to the specific adaptive changes needed by today’s biotechnologist. Ancient polymerases, for example, were most likely *not* evolving to become resistant to heparin, a target of one of Holliger’s selections. Therefore, the rationale for exploiting “evolutionary guidance” is more subtle.

A REAP analysis identifies sites that have been historically involved in *some* adaptive event. Because *some* changes are involved, the amino acid at the site cannot be *absolutely* required for core function. Conversely, the REAP-identified sites are not likely to be those whose amino acids *never* have a phenotypic impact. The rationale being that sites that have in the past been involved in an adaptive event without losing core function are sites that might be productively examined to identify sites that might adapt the protein to the *new*, biotechnologist-demanded, function.

Thus, the rationale behind REAP is the hypothesis that the most productive sites to replace in a protein engineering experiment are neither sites whose amino acids contribute to a core function (as indicated by their absolute conservation) nor sites in which the choice of amino acid is incidental to function (as indicated by their easy variability). By identifying sites for which replacement might have phenotypic impact without destroying core function, REAP is proposed to have an advantage compared to other methods in the generation of libraries with productively altered behaviors. The advantage of the REAP approach relies on the fact that nature has already tested several amino acid sites, and these modifications on these sites produce enzymes that retain the original activity. Searching for new variants in a REAP library gives the advantage of having several parent enzymes.

Thus, the design of a high fidelity DNA polymerase from a medium fidelity polymerase is largely beyond current structure theory. This makes it impossible to get polymerases with the desired high level behaviors from fully guided protein engineering. As a consequence, many investigators use protein engineering to select for polymerases with certain properties improved with respect to a desired function, starting from libraries of polymerase variants. The directed evolution approach is today considered by many to be the method of choice for protein engineering (Bornscheuer and Pohl, 2001; Yuan et al., 2005; Leemhuis et al., 2009; Turner, 2009).

#### Compartmentalization

Directed evolution requires the connecting of a phenotype with a genotype in a way that allows only genes that confer a desired phenotype to be propagated. This can be done in many ways. One method is compartmentalized self replication (CSR). Developed by Tawfik and Griffiths (1998), CSR holds proteins and genes together in water droplets suspended in oil emulsions. These generally receive the gene-protein pair from a single *E. coli* cell that is encapsulated within individual droplets (Tawfik and Griffiths, 1998). When the protein is a polymerase variant, its gene is copied only if that variant is active under the conditions of the evolution experiment.

Compartmentalized self replication was first applied to the directed evolution of DNA polymerases by Ghadessy et al. (2001). Here, a library of polymerase genes was delivered in plasmids to create clones in *E. coli* cells. These cells were dispersed into emulsified water droplets containing the primers and buffers needed to perform a PCR amplification of the polymerase gene. Approximately ∼10^8^-10^9^ compartments are formed per milliliter of emulsion; ideally, each compartment contains a single variant. PCR cycling is then performed, with the first heat step lysing the *E. coli* cell to present its expressed thermostable polymerase and its encoding plasmids to the primers. Lysis of the cells then delivers polymerase variants expressed inside of the cells to the buffer, which contains the necessary components for PCR. The polymerase variants and the contents of the buffer remain encapsulated during the PCR cycling.

**FIGURE 3:**
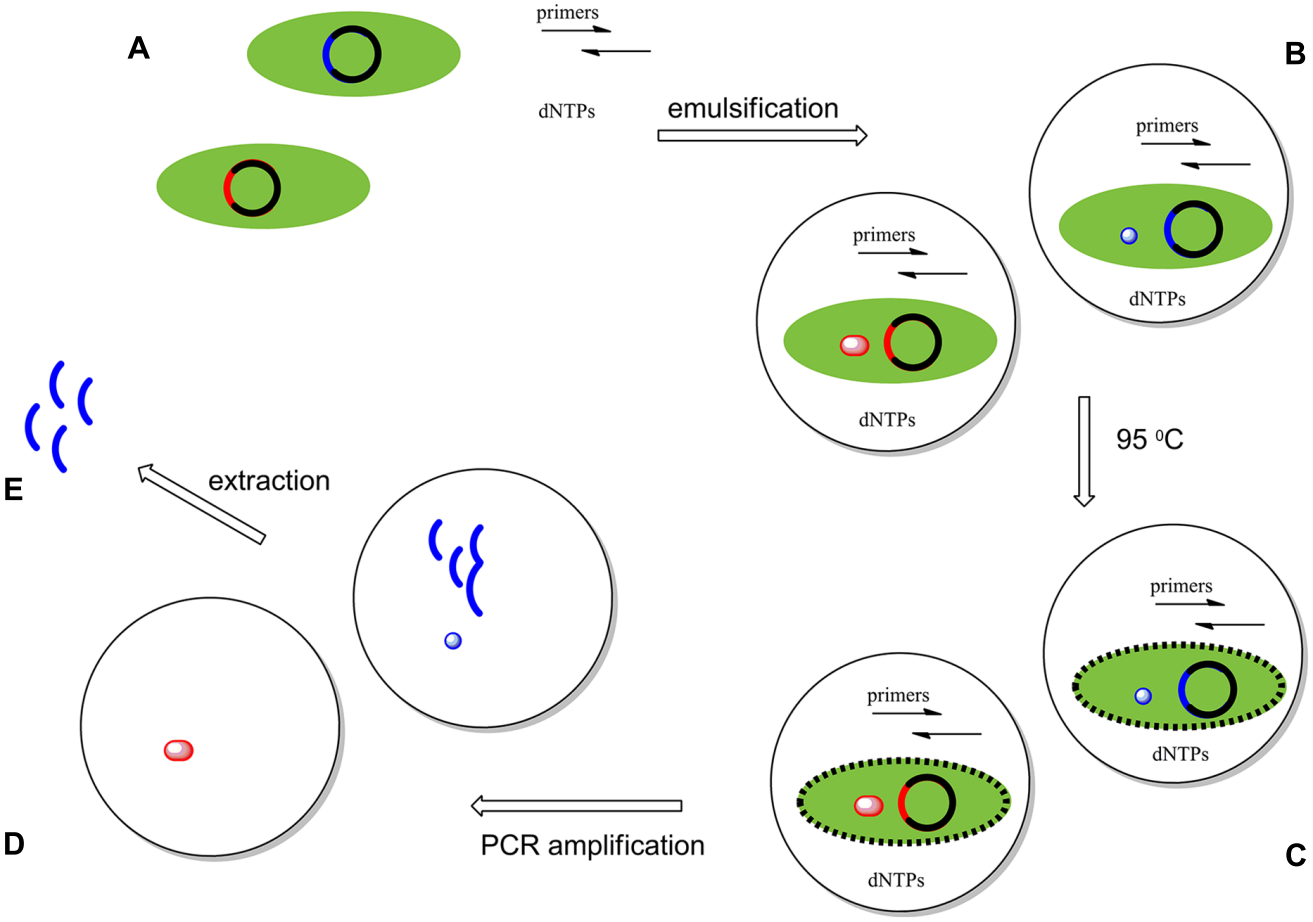
**Compartmentalized self replication (CSR) system experiments start with the creation of a library of genes encoding variants of a polymerase.** Members of this library are introduced into *E. coli* cells by electroporation. Here, just two variant genes (red and blue) are represented. These genes drive the expression of mutant polymerases in each *E. coli* cell, each of which is isolated in its own water-in-oil-emulsion droplet. **(B)** The first cycle of PCR breaks the cell wall of the *E. coli*, exposing the expressed polymerase molecules and their gene to the contents of a water droplet containing all of the necessary components necessary for a PCR amplification: (i) primers, (ii) dNTPs, (iii) a mutated gene of the polymerase, and (iv) the enzyme expressed by this gene **(C)**. During PCR, any polymerases active under the selective pressure (blue) amplify their respective genes, enriching the pool of mutants having the desired properties; inactive polymerases (red) fail to do so **(D)**. The emulsion is then broken and the amplified genes enriched in those encoding polymerases having the desired behaviors are extracted and inserted in a plasmid vector [circular DNA; **E**]. These then enter the cycle of selection again **(A)**. After repeating these cycles an enriched pool of variants of the original gene are produced.

Polymerases that functioned under the conditions imposed by the experiment were able to make copies of only their own genes. After 20 rounds or more of PCR, the emulsions are broken to give a pool of PCR products enriched in the genes that encoded the selected polymerase variants. These genes could be used directly, or be introduced in cells for another round of selection. This process is shown schematically in **Figure 3**. With iteration, this process mimics natural evolution, except that the selective pressures applied come from the bioengineer, rather than from Nature.

Phage display is an alternative way to connect genotype and phenotype. In it, a polymerase is linked to its encoding gene in a single viral particle. The protein of interest is co-expressed on the coat of a virus, linking genotype to phenotype. The Romesberg laboratory has been especially active in generating polymerase variants using this approach (Xia et al., 2002; Leconte et al., 2005, 2010).

## EXAMPLES OF MODIFIED POLYMERASES

### CONVERTING A DNA POLYMERASE TO AN RNA POLYMERASE

Misincorporation by a DNA polymerase through the incorporation of ribonucleoside triphosphates, rather than deoxynucleoside triphosphates, would circumvent the normal pathways in living cells. Accordingly, all DNA polymerases utilize a common mechanism to avoid misincorporation of ribonucleotides by a single active site residue known as the “steric gate” (Joyce, 1997; Gardner and Jack, 1999; Brown and Suo, 2011). Mutations in the steric gate alone are sufficient to render the DNA polymerase able to incorporate nucleoside triphosphates. Yet, products lengths have not exceeded 58 nucleotides and generally result in short termination sequences stalling at +6–7 nucleotides (Gao et al., 1997; Gardner and Jack, 1999; Patel and Loeb, 2000b; Xia et al., 2002; Yang et al., 2002; Ong et al., 2006; McCullum and Chaput, 2009; Brown et al., 2010; Staiger and Marx, 2010; Brown and Suo, 2011). Recently, Cozens et al. (2012) discovered a single amino acid mutation (E664K) in the DNA polymerase from *Thermococcus gorgonarius* that in conjunction with a “steric gate mutation” produced a DNA polymerase capable of synthesizing long RNAs, up 1.7 kb.

Using phage display the Romesberg laboratory has evolved a DNA polymerase [the Stoffel fragment (Sf) of Taq polymerase] into a RNA polymerase. With just five mutations, one of them the “steric gate mutation” (E615G in Taq) the DNA polymerase was able to incorporate ribonucleotides triphosphates (rNTPs) with rates increased by 10^3^–10^4^ fold compared to the wild type polymerase (Xia et al., 2002). The Holliger laboratory known for the use of the CSR approach has produced a variant of *Taq* polymerase that can incorporate both dNTPs and rNTPs; this variant has only four mutations one of them the “steric gate mutation” mentioned in the previous example (Ong et al., 2006).

### DNA POLYMERASES ABLE TO BYPASS DEFECTS

d’Abbadie et al. (2007) shuﬄed the genes of the polymerases from three *Thermus* species (*aquaticus*, *thermophilus,* and *flavus*) to generate libraries to start a directed evolution experiment to identify DNA polymerases that can extend single, double and quadruple mismatches, process non-canonical primer-template duplexes, and bypass hydantoins and abasic sites (d’Abbadie et al., 2007). They applied these to PCR-amplify cave bear DNA from remains ca. 50 000 years old. These experiments showed that the polymerases obtained by directed evolution applied to these libraries outperformed Taq DNA polymerase and were, therefore, better able to solve a biotechnological problem, here, the sequencing of ancient damaged genomes.

### DNA POLYMERASES ABLE TO ACCEPT EXPANDED GENETIC ALPHABETS

**FIGURE 4:**
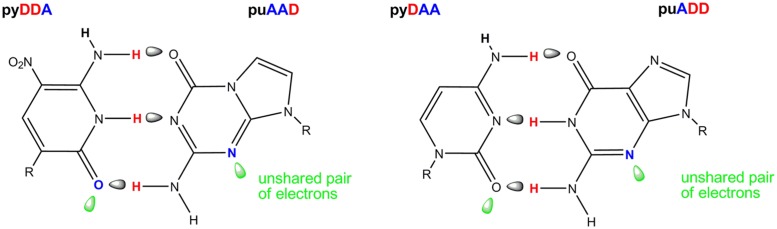
**The Z:P pair from an artificially expanded genetic information system (AEGIS, left) showing in green the orbitals containing the unshared electron pairs presented to the minor groove of DNA.** The natural C:G pair (right) showing these unshared pairs of electrons in the standard C:G pair. The pattern of hydrogen bonding is indicated A, hydrogen bond acceptor and D, hydrogen bond donor.

One of the AEGIS base pair created in our laboratories is formed between the nucleotides trivially called Z and P (**Figure 4**). The Z:P pair has a standard Watson-Crick geometry joined by three hydrogen bonds, differing from the standard C:G pair in the arrangement of donor and acceptor groups that form the connecting hydrogen bonds. Both nucleobases place electron density into the minor groove, a density that can accept a hydrogen bond from a polymerase (Geyer et al., 2003). These features allow polymerases to accept dZTP and dPTP as substrates to form duplexes containing Z:P pairs in primer extension reactions, PCR and nested PCR architectures.

In order to improve polymerases able to accept these AEGIS components, we did a selection among Δ(1-279) *Taq* using CSR. Two of the best variants identified: variant (M444V/P527A/D551E/E832V) and variant (N580S/L628V/E832V) showed to pause less when challenged *in vitro* to incorporate dZTP opposite P in a template (Laos et al., 2013). Interestingly, our library was created by introducing random mutations on the *Taq* gene, but the outcome of the selection produced variants which contain several sites that have displayed heterotachy (different rates of change) in their natural history (Lopez et al., 2002). Heterotachy is a sequence pattern such that the rate of evolution acting at an individual site can be slow in one portion of the phylogeny while the rate at the same site can be rapid in a different portion of the phylogeny. Such patterns arise from shifts in the selective constraints acting at individual sites throughout the evolutionary history of a gene family, and by extension, the precise biomolecular behaviors of the homologous proteins are not identical across the phylogeny (Chen et al., 2010; Cole and Gaucher, 2011) suggesting that these sites were involved in an adaptive change in natural polymerase evolution.

The Romesberg laboratory, using phage display, has produced polymerases having an improved ability to incorporate the self-pairing hydrophobic nucleobases analog propynylisocarbostyril (PICS; Leconte et al., 2005).

Loakes et al. (2009) have produced a novel polymerase product of the shuﬄing of polymerases from family A (*Taq* from *T. aquaticus*, *Tth* from *T. thermophilus*, and *Tfl* from *T. flavus*) all of them from the genus *Thermus* and selected by CSR.

### POLYMERASES FOR SEQUENCING BY SYNTHESIS METHODS

Sequencing by synthesis (SBS) is a promising next-generation DNA sequencing approach. There are currently several commercial instruments that are offered in the market. Some of the common features of these products are the use of solid phase chemistry to amplify the initial sample and the use of reversible terminators. Reversible terminators are nucleotides that generally have two modifications: one at the 3′OH and the other is either at the 5 or 7 position of the nucleobase. The 3′ hydroxyl position has a cleavable moiety that terminates the polymerase extension reaction after a single-base incorporation. Yet, some reversible terminators do not have a modification on the 3′ hydroxyl like some scarless photocleavable terminator of LaserGen (Wu et al., 2007), the virtual terminator of Helicos BioSciences (Bowers et al., 2009) and the more recently reported Lightning Terminators^TM^ developed in New England Biolabs (Gardner et al., 2012).

The other modification is at the C-5 of pyrimidines or the N-7 position of purines and consist of a fluorescent molecule that is used as a reporter for each of the individual bases. The C-5 and N-7 positions are used because these positions point away from the catalytic pocket of the enzyme. Gardner and Jack (1999) studied variants of Vent DNA polymerase from the hyperthermophilic archaeon *Thermococcus litoralis*. They studied variants on a Tyrosine residue that is highly conserved on family B and was proposed to act as a steric gate (Gardner and Jack, 1999).

The Romesberg laboratories have found polymerases having an improved ability to incorporate modified dUTP with a fluorophore (dUTP-Fl) that can be used for SBS. Leconte et al. (2010) generated a library of Sf, which is *Taq* DNA polymerase minus the first 289 amino acids. This fragment conserves the polymerase activity but lacks the exonuclease domain. The library was done by shuﬄing the genes of six homologous polymerases: *Thermus aquaticus*; *Thermus thermophilus*; *Thermus caldophilus*; *Thermus filiformis*; *Spirochaeta thermophila*; *and Thermomicrobium roseum*. The three most active polymerase mutants were: Sf168 (with 19 mutations); Sf197 (with 14 mutations). These mutants had between 10 to 50-fold increase in efficiency for dUTP-Fl incorporation compared with wild type Sf (Leconte et al., 2010).

Our laboratories have produced a variant of Taq polymerase using an evolutionary approach to design a polymerase library and then screen a relatively small library for polymerases able to accept unnatural triphosphates modified on their sugar units. Using REAP, they identified 35 sites having heterotachous behavior, after filtering for sites where additional information from evolutionary history, structural biology, and experiments was exploited. They then asked which replacements improve the ability of *Taq* polymerase to accept reversible terminating triphosphates, where the 3′-OH unit of the nucleoside triphosphate had been replaced by an -ONH_2_ unit, which prevents continued primer extensions. A single modification (L616A) appears to open space behind Phe-667, allowing the enzyme to accommodate a larger 3′-substituent (Chen et al., 2010).

The Holliger lab, when selecting for variants that accepted 2′-deoxycytidine derivatives carrying appended Cy3- and Cy5 fluorescent dyes, recovered variants of *Pfu* DNA polymerase each having two to six amino acid replacements (Ramsay et al., 2010).

## COMMERCIAL APPLICATIONS

The number of commercial applications for non-standard nucleotides is large and growing, implying a growing need for engineered polymerases. This review cannot describe all of the potential commercial applications, but a brief summary of those that already exist indicates their scope. For example, Sherrill et al. (2004) used isoC and isoG modified with reporter molecules to develop an assay to detect both RNA and DNA. These modified nucleic acids (isoC and isoG) are also used to quantify levels of HIV and hepatitis viruses in patients (Collins et al., 1997; Elbeik et al., 2004a,b). isoC and isoG are also used to diagnose a panel of respiratory diseases (Nolte et al., 2007).

Sequencing by synthesis technology relies on nucleoside derivatives that are modified in two ways, first with a fluorescent tag, and then (usually) with a reversibly terminating blocking group (Fedurco et al., 2006; Turcatti et al., 2008). Different modified polymerases, which are commercially available have been suggested to improve the procedure (Aird et al., 2011; Fisher et al., 2011; Quail et al., 2012). Real-time sequencing also requires polymerases that accept nucleoside derivatives (Eid et al., 2009).

At least one polymerase obtained by directed evolution is commercially available; it was selected to the ability to incorporate dZTP opposite dP in a template, and is available through Firebird Biomolecular Sciences LLC (www.firebirdbio.com).

## CONCLUSION AND PERSPECTIVES

The demand for polymerases capable of incorporating unnatural nucleotides is certain to grow as the interest to build modified DNA structures continues, including alternative genetic alphabets (Geyer et al., 2003), highly tagged substrates (Hollenstein et al., 2009), modified backbones (Pinheiro et al., 2012), and other unusual structures (Fa et al., 2004; Leconte et al., 2005; Hirao et al., 2007).

The literature teaches that in some cases, simple downstream screening can obtain polymerases with the needed properties. This is illustrated by efforts by Tabor and Richardson (1995), Gardner and Jack (1999), and Chen et al. (2010) to name a few examples. In these cases to create polymerases that accept various 3′-terminating groups. Their combination of structural biology and evolutionary biology analyses were sufficiently powerful to ensure that regions of sequence space small enough to be screened containing polymerases having the desired properties. In each case, screening began with a relatively small number of variants extracted from the sequence space local around a deftly chosen parent, allowing to get useful enzymes by inspection. However, as a result of the large space sequence space of proteins, examples of success in designing polymerases are not frequent, and are rarely (if ever) *de novo*.

In our experience, the outcome of directed evolution experiments can be explained by the analysis of the evolutionary history of the protein; in this case we found the heterotachy pattern. The heterotachy analysis was originally used to elaborate a small library and screen for variants of *Taq* polymerase (Chen et al., 2010). Later we found several of the substitutions recovered in our directed evolution experiment with *Taq* polymerase had this pattern.

It is interesting to note that some mutations reported in the literature by other research groups happen in amino acid sites considered to be heterotachous by our analysis. One particular amino acid change found by our selection of polymerases better able to synthesize duplexes containing Z:P pairs (D578N) occurred in a site that underwent substitution in the CSR experiment that obtained a *Taq* variant resistant to heparin inhibition (D578G; Ghadessy et al., 2001). Remarkably, position 614 on *Taq* polymerase has been reported at least three times as the outcome of directed evolution experiments (Patel et al., 2001; Xia et al., 2002; Fa et al., 2004). Other amino acid sites from *Taq* polymerase considered heterotachous and reported on directed evolution experiments are: D144, F598 (Ghadessy et al., 2004), A597, A600, E615 (Xia et al., 2002), and L616 (Patel et al., 2001). This provides support for the general hypothesis behind REAP, that sites involved in adaptation to one environmental novelty might also help adaptation to environmental novelties more generally.

We believe that the recapitulation of the natural history of proteins reflects the fact that the sites can be changed to meet new challenges presented to polymerases without damaging the catalytic power or fidelity of the proteins. The observations in the literature underline the importance of understanding the evolution of polymerases in designing libraries to better explore their sequence space. It will be interesting to further study the outcome of contemporary *in vitro* selection experiments and how they recapitulate.

For less effectively constructed libraries of variants, including those generated by shuﬄing and by undirected mutagenesis, various selection tools stand to pick up where screening cannot possibly go. Here, CSR and phage display have been especially useful. These have yielded polymerases that support the copying of entirely different genetic systems (Pinheiro et al., 2012).

Although some of the mutations found to be useful for altering polymerases to accept unnatural nucleotides fall on or near the conserved motifs and could potentially be rationalized, there are still a number of mutations that cannot be easily explained and their effect could be subtle.

Other approaches have been found to be useful for the evolution of other enzyme systems. For example, neutral drift libraries (Amitai et al., 2007; Bloom et al., 2007a,b), have yet to be applied as starting points for directed evolution experiments with DNA polymerases.

Further, we (and many others) are seeking to develop living systems that implement a “synthetic biology” based on unnatural DNA analogs. These have the potential for being “biosafe” platforms for artificial metabolisms, fermentations, diagnostics, and therapeutic tools, *inter alia* (Schmidt, 2010).

## Conflict of Interest Statement

The authors declare that the research was conducted in the absence of any commercial or financial relationships that could be construed as a potential conflict of interest.

## Footnotes

1 N is any nucleotide.

2 Y is a pyrimidine.
